# Supplementary material for: HIVprotI: an integrated web based platform for prediction and design of HIV proteins inhibitors
Source: J Cheminform. 2018 Mar 9;10:12. doi: 10.1186/s13321-018-0266-y (PMC5845081; doi:10.1186/s13321-018-0266-y)
Supplement: Supplementary file 2 — Additional file 2. Source code of HIVProtI web server. [file 13321_2018_266_MOESM2_ESM.zip › HIVprotI_Source-code/hivprotI/weka-3-6-6/doc/weka/core/xml/XMLSerialization.html]

XMLSerialization


---


|  |  |  |
| --- | --- | --- |
| **Overview** \| **Package** \| **Class** \| **Tree** \| **Deprecated** \| **Index** \| **Help** \| **Weka's home** | |  |
| **PREV CLASS**   **NEXT CLASS** | **FRAMES**    **NO FRAMES**     **All Classes** |  |
| SUMMARY: NESTED \| FIELD \| CONSTR \| METHOD | DETAIL: FIELD \| CONSTR \| METHOD |  |


---


## weka.core.xml Class XMLSerialization

```
java.lang.Object
  weka.core.xml.XMLSerialization
```

**All Implemented Interfaces:**: RevisionHandler

**Direct Known Subclasses:**: XMLBasicSerialization

---

``` public class XMLSerialization extends java.lang.Object implements RevisionHandler ```

With this class objects can be serialized to XML instead into a binary
format. It uses introspection (cf. beans) to retrieve the data from the
given object, i.e. it can only access beans-conform fields automatically.

The generic approach of writing data as XML can be overriden by adding
custom methods for reading/writing in a derived class
(cf. `m_Properties`, `m_CustomMethods`).  
Custom read and write methods must have the same signature (and also be
`public`!) as the `readFromXML` and `writeToXML`
methods. Methods that apply to the naming rule `read + property name`
are added automatically to the list of methods by the method
`XMLSerializationMethodHandler.addMethods(...)`.

Other properties that are not conform the bean set/get-methods have to be
processed manually in a derived class (cf. `readPostProcess(Object)`,
`writePostProcess(Object)`).

For a complete XML serialization/deserialization have a look at the
`KOML` class.

If a stored class has a constructor that takes a String to initialize
(e.g. String or Double) then the content of the tag will used for the
constructor, e.g. from

```
<object name="name" class="String" primitive="no">Smith</object>
```

"Smith" will be used to instantiate a String object as constructor argument.

**Version:**
:   $Revision: 1.16 $

**Author:**
:   FracPete (fracpete at waikato dot ac dot nz)

**See Also:**: `KOML`, `fromXML(Document)`, `toXML(Object)`, `m_Properties`, `m_CustomMethods`, `readPostProcess(Object)`, `writePostProcess(Object)`, `readFromXML(Element)`, `writeToXML(Element, Object, String)`

---

| **Field Summary** | |
| --- | --- |
| `static java.lang.String` | `ATT_ARRAY`             the tag whether array or not (yes/no) |
| `static java.lang.String` | `ATT_ARRAY_DEFAULT`             default value for attribute ATT\_ARRAY |
| `static java.lang.String` | `ATT_CLASS`             the tag for the class |
| `static java.lang.String` | `ATT_NAME`             the tag for the name |
| `static java.lang.String` | `ATT_NULL`             the tag whether null or not (yes/no) |
| `static java.lang.String` | `ATT_NULL_DEFAULT`             default value for attribute ATT\_NULL |
| `static java.lang.String` | `ATT_PRIMITIVE`             the tag whether primitive or not (yes/no) |
| `static java.lang.String` | `ATT_PRIMITIVE_DEFAULT`             default value for attribute ATT\_PRIMITIVE |
| `static java.lang.String` | `ATT_VERSION`             the version attribute |
| `static java.lang.String` | `DOCTYPE`             the DOCTYPE for the serialization |
| `static java.lang.String` | `ROOT_NODE`             the root node of the XML document |
| `static java.lang.String` | `TAG_OBJECT`             the tag for an object |
| `static java.lang.String` | `VAL_NO`             the value "no" for the primitive and array attribute |
| `static java.lang.String` | `VAL_ROOT`             the value of the name for the root node |
| `static java.lang.String` | `VAL_YES`             the value "yes" for the primitive and array attribute |


**Constructor Summary**

`XMLSerialization()`
  
          initializes the serialization


| **Method Summary** | |
| --- | --- |
| `void` | `clear()`             generates internally a new XML document and clears also the IgnoreList and the mappings for the Read/Write-Methods |
| `java.lang.Object` | `fromXML(org.w3c.dom.Document document)`             returns the given DOM document as an instance of the specified class |
| `java.lang.String` | `getRevision()`             Returns the revision string. |
| `java.lang.String` | `getVersion()`             returns the WEKA version with which the serialized object was created |
| `static void` | `main(java.lang.String[] args)`             for testing only. |
| `java.lang.Object` | `read(java.io.File file)`             parses the given file and returns a DOM document |
| `java.lang.Object` | `read(java.io.InputStream stream)`             parses the given stream and returns a DOM document |
| `java.lang.Object` | `read(java.io.Reader reader)`             parses the given reader and returns a DOM document |
| `java.lang.Object` | `read(java.lang.String xml)`             parses the given XML string (can be XML or a filename) and returns an Object generated from the representation |
| `boolean` | `readBooleanFromXML(org.w3c.dom.Element node)`             builds the primitive from the given DOM node. |
| `byte` | `readByteFromXML(org.w3c.dom.Element node)`             builds the primitive from the given DOM node. |
| `char` | `readCharFromXML(org.w3c.dom.Element node)`             builds the primitive from the given DOM node. |
| `double` | `readDoubleFromXML(org.w3c.dom.Element node)`             builds the primitive from the given DOM node. |
| `float` | `readFloatFromXML(org.w3c.dom.Element node)`             builds the primitive from the given DOM node. |
| `java.lang.Object` | `readFromXML(org.w3c.dom.Element node)`             builds the object from the given DOM node. |
| `java.lang.Object` | `readFromXML(java.lang.Object o, java.lang.String name, org.w3c.dom.Element child)`             adds the specific node to the object via a set method |
| `int` | `readIntFromXML(org.w3c.dom.Element node)`             builds the primitive from the given DOM node. |
| `long` | `readLongFromXML(org.w3c.dom.Element node)`             builds the primitive from the given DOM node. |
| `short` | `readShortFromXML(org.w3c.dom.Element node)`             builds the primitive from the given DOM node. |
| `XMLDocument` | `toXML(java.lang.Object o)`             extracts all accesible properties from the given object |
| `void` | `write(java.io.File file, java.lang.Object o)`             writes the given object into the file |
| `void` | `write(java.io.OutputStream stream, java.lang.Object o)`             writes the given object into the stream |
| `void` | `write(java.lang.String file, java.lang.Object o)`             writes the given object into the file |
| `void` | `write(java.io.Writer writer, java.lang.Object o)`             writes the given object into the writer |
| `org.w3c.dom.Element` | `writeToXML(org.w3c.dom.Element parent, java.lang.Object o, java.lang.String name)`             adds the given Object to a DOM structure. |

**Methods inherited from class java.lang.Object**

`equals, getClass, hashCode, notify, notifyAll, toString, wait, wait, wait`

**Field Detail**

### TAG\_OBJECT

```
public static final java.lang.String TAG_OBJECT
```

:   the tag for an object

    **See Also:**: Constant Field Values

---


### ATT\_VERSION

```
public static final java.lang.String ATT_VERSION
```

:   the version attribute

    **See Also:**: Constant Field Values

---


### ATT\_NAME

```
public static final java.lang.String ATT_NAME
```

:   the tag for the name

    **See Also:**: Constant Field Values

---


### ATT\_CLASS

```
public static final java.lang.String ATT_CLASS
```

:   the tag for the class

    **See Also:**: Constant Field Values

---


### ATT\_PRIMITIVE

```
public static final java.lang.String ATT_PRIMITIVE
```

:   the tag whether primitive or not (yes/no)

    **See Also:**: Constant Field Values

---


### ATT\_ARRAY

```
public static final java.lang.String ATT_ARRAY
```

:   the tag whether array or not (yes/no)

    **See Also:**: Constant Field Values

---


### ATT\_NULL

```
public static final java.lang.String ATT_NULL
```

:   the tag whether null or not (yes/no)

    **See Also:**: Constant Field Values

---


### VAL\_YES

```
public static final java.lang.String VAL_YES
```

:   the value "yes" for the primitive and array attribute

    **See Also:**: Constant Field Values

---


### VAL\_NO

```
public static final java.lang.String VAL_NO
```

:   the value "no" for the primitive and array attribute

    **See Also:**: Constant Field Values

---


### VAL\_ROOT

```
public static final java.lang.String VAL_ROOT
```

:   the value of the name for the root node

    **See Also:**: Constant Field Values

---


### ROOT\_NODE

```
public static final java.lang.String ROOT_NODE
```

:   the root node of the XML document

    **See Also:**: Constant Field Values

---


### ATT\_PRIMITIVE\_DEFAULT

```
public static final java.lang.String ATT_PRIMITIVE_DEFAULT
```

:   default value for attribute ATT\_PRIMITIVE

    **See Also:**: `ATT_PRIMITIVE`, Constant Field Values

---


### ATT\_ARRAY\_DEFAULT

```
public static final java.lang.String ATT_ARRAY_DEFAULT
```

:   default value for attribute ATT\_ARRAY

    **See Also:**: `ATT_ARRAY`, Constant Field Values

---


### ATT\_NULL\_DEFAULT

```
public static final java.lang.String ATT_NULL_DEFAULT
```

:   default value for attribute ATT\_NULL

    **See Also:**: `ATT_NULL`, Constant Field Values

---


### DOCTYPE

```
public static final java.lang.String DOCTYPE
```

:   the DOCTYPE for the serialization


**Constructor Detail**

### XMLSerialization

```
public XMLSerialization()
                 throws java.lang.Exception
```

:   initializes the serialization

    **Throws:**: `java.lang.Exception` - if initialization fails


**Method Detail**

### clear

```
public void clear()
           throws java.lang.Exception
```

:   generates internally a new XML document and clears also the IgnoreList and
    the mappings for the Read/Write-Methods

    :   **Throws:**: `java.lang.Exception` - if something goes wrong

---


### getVersion

```
public java.lang.String getVersion()
```

:   returns the WEKA version with which the serialized object was created

    :   **Returns:**: the current version **See Also:**: `Version`

---


### writeToXML

```
public org.w3c.dom.Element writeToXML(org.w3c.dom.Element parent,
                                      java.lang.Object o,
                                      java.lang.String name)
                               throws java.lang.Exception
```

:   adds the given Object to a DOM structure.
    (only public due to reflection).  
    **Note:** `overrideClassname(Object)` is not invoked in case of
    arrays, since the array class could be a superclass, whereas the elements of
    the array can be specialized subclasses. In case of an array the method
    `overrideClassname(String)` is invoked, which searches for an
    exact match of the classname in the override hashtable.

    :   **Parameters:**: `parent` - the parent of this object, e.g. the class this object is a member of: `o` - the Object to describe in XML: `name` - the name of the object **Returns:**: the node that was created **Throws:**: `java.lang.Exception` - if the DOM creation fails **See Also:**: `overrideClassname(Object)`, `overrideClassname(String)`, `m_ClassnameOverride`

---


### toXML

```
public XMLDocument toXML(java.lang.Object o)
                  throws java.lang.Exception
```

:   extracts all accesible properties from the given object

    :   **Parameters:**: `o` - the object to turn into an XML representation **Returns:**: the generated DOM document **Throws:**: `java.lang.Exception` - if XML generation fails

---


### readBooleanFromXML

```
public boolean readBooleanFromXML(org.w3c.dom.Element node)
                           throws java.lang.Exception
```

:   builds the primitive from the given DOM node.

    :   **Parameters:**: `node` - the associated XML node **Returns:**: the primitive created from the XML description **Throws:**: `java.lang.Exception` - if instantiation fails

---


### readByteFromXML

```
public byte readByteFromXML(org.w3c.dom.Element node)
                     throws java.lang.Exception
```

:   builds the primitive from the given DOM node.

    :   **Parameters:**: `node` - the associated XML node **Returns:**: the primitive created from the XML description **Throws:**: `java.lang.Exception` - if instantiation fails

---


### readCharFromXML

```
public char readCharFromXML(org.w3c.dom.Element node)
                     throws java.lang.Exception
```

:   builds the primitive from the given DOM node.

    :   **Parameters:**: `node` - the associated XML node **Returns:**: the primitive created from the XML description **Throws:**: `java.lang.Exception` - if instantiation fails

---


### readDoubleFromXML

```
public double readDoubleFromXML(org.w3c.dom.Element node)
                         throws java.lang.Exception
```

:   builds the primitive from the given DOM node.

    :   **Parameters:**: `node` - the associated XML node **Returns:**: the primitive created from the XML description **Throws:**: `java.lang.Exception` - if instantiation fails

---


### readFloatFromXML

```
public float readFloatFromXML(org.w3c.dom.Element node)
                       throws java.lang.Exception
```

:   builds the primitive from the given DOM node.

    :   **Parameters:**: `node` - the associated XML node **Returns:**: the primitive created from the XML description **Throws:**: `java.lang.Exception` - if instantiation fails

---


### readIntFromXML

```
public int readIntFromXML(org.w3c.dom.Element node)
                   throws java.lang.Exception
```

:   builds the primitive from the given DOM node.

    :   **Parameters:**: `node` - the associated XML node **Returns:**: the primitive created from the XML description **Throws:**: `java.lang.Exception` - if instantiation fails

---


### readLongFromXML

```
public long readLongFromXML(org.w3c.dom.Element node)
                     throws java.lang.Exception
```

:   builds the primitive from the given DOM node.

    :   **Parameters:**: `node` - the associated XML node **Returns:**: the primitive created from the XML description **Throws:**: `java.lang.Exception` - if instantiation fails

---


### readShortFromXML

```
public short readShortFromXML(org.w3c.dom.Element node)
                       throws java.lang.Exception
```

:   builds the primitive from the given DOM node.

    :   **Parameters:**: `node` - the associated XML node **Returns:**: the primitive created from the XML description **Throws:**: `java.lang.Exception` - if instantiation fails

---


### readFromXML

```
public java.lang.Object readFromXML(java.lang.Object o,
                                    java.lang.String name,
                                    org.w3c.dom.Element child)
                             throws java.lang.Exception
```

:   adds the specific node to the object via a set method

    :   **Parameters:**: `o` - the object to set a property: `name` - the name of the object for which to set a property (only for information reasons): `child` - the value of the property to add **Returns:**: the provided object, but augmented by the child **Throws:**: `java.lang.Exception` - if something goes wrong

---


### readFromXML

```
public java.lang.Object readFromXML(org.w3c.dom.Element node)
                             throws java.lang.Exception
```

:   builds the object from the given DOM node.
    (only public due to reflection)

    :   **Parameters:**: `node` - the associated XML node **Returns:**: the instance created from the XML description **Throws:**: `java.lang.Exception` - if instantiation fails

---


### fromXML

```
public java.lang.Object fromXML(org.w3c.dom.Document document)
                         throws java.lang.Exception
```

:   returns the given DOM document as an instance of the specified class

    :   **Parameters:**: `document` - the parsed DOM document representing the object **Returns:**: the XML as object **Throws:**: `java.lang.Exception` - if object instantiation fails

---


### read

```
public java.lang.Object read(java.lang.String xml)
                      throws java.lang.Exception
```

:   parses the given XML string (can be XML or a filename) and returns an
    Object generated from the representation

    :   **Parameters:**: `xml` - the xml to parse (if "xml" is not found then it is considered a file) <DT**Returns:**: the generated instance **Throws:**: `java.lang.Exception` - if something goes wrong with the parsing

---


### read

```
public java.lang.Object read(java.io.File file)
                      throws java.lang.Exception
```

:   parses the given file and returns a DOM document

    :   **Parameters:**: `file` - the XML file to parse **Returns:**: the parsed DOM document **Throws:**: `java.lang.Exception` - if something goes wrong with the parsing

---


### read

```
public java.lang.Object read(java.io.InputStream stream)
                      throws java.lang.Exception
```

:   parses the given stream and returns a DOM document

    :   **Parameters:**: `stream` - the XML stream to parse **Returns:**: the parsed DOM document **Throws:**: `java.lang.Exception` - if something goes wrong with the parsing

---


### read

```
public java.lang.Object read(java.io.Reader reader)
                      throws java.lang.Exception
```

:   parses the given reader and returns a DOM document

    :   **Parameters:**: `reader` - the XML reader to parse **Returns:**: the parsed DOM document **Throws:**: `java.lang.Exception` - if something goes wrong with the parsing

---


### write

```
public void write(java.lang.String file,
                  java.lang.Object o)
           throws java.lang.Exception
```

:   writes the given object into the file

    :   **Parameters:**: `file` - the filename to write to: `o` - the object to serialize as XML **Throws:**: `java.lang.Exception` - if something goes wrong with the parsing

---


### write

```
public void write(java.io.File file,
                  java.lang.Object o)
           throws java.lang.Exception
```

:   writes the given object into the file

    :   **Parameters:**: `file` - the filename to write to: `o` - the object to serialize as XML **Throws:**: `java.lang.Exception` - if something goes wrong with the parsing

---


### write

```
public void write(java.io.OutputStream stream,
                  java.lang.Object o)
           throws java.lang.Exception
```

:   writes the given object into the stream

    :   **Parameters:**: `stream` - the filename to write to: `o` - the object to serialize as XML **Throws:**: `java.lang.Exception` - if something goes wrong with the parsing

---


### write

```
public void write(java.io.Writer writer,
                  java.lang.Object o)
           throws java.lang.Exception
```

:   writes the given object into the writer

    :   **Parameters:**: `writer` - the filename to write to: `o` - the object to serialize as XML **Throws:**: `java.lang.Exception` - if something goes wrong with the parsing

---


### main

```
public static void main(java.lang.String[] args)
                 throws java.lang.Exception
```

:   for testing only. if the first argument is a filename with ".xml"
    as extension it tries to generate an instance from the XML description
    and does a `toString()` of the generated object.

    :   **Throws:**: `java.lang.Exception`

---


### getRevision

```
public java.lang.String getRevision()
```

:   Returns the revision string.

    :   **Specified by:**: `getRevision` in interface `RevisionHandler`
    :   **Returns:**: the revision


---


|  |  |  |
| --- | --- | --- |
| **Overview** \| **Package** \| **Class** \| **Tree** \| **Deprecated** \| **Index** \| **Help** \| **Weka's home** | |  |
| **PREV CLASS**   **NEXT CLASS** | **FRAMES**    **NO FRAMES**     **All Classes** |  |
| SUMMARY: NESTED \| FIELD \| CONSTR \| METHOD | DETAIL: FIELD \| CONSTR \| METHOD |  |


---
